# Histological, Biochemical, and Hematological Effects of Goniothalamin on Selective Internal Organs of Male* Sprague-Dawley* Rats

**DOI:** 10.1155/2019/6493286

**Published:** 2019-04-23

**Authors:** Fahmi Kaid, A. M. Alabsi, Nashwan Alafifi, Rola Ali-Saeed, May Ameen Al-koshab, Anand Ramanathan, A. M. Ali

**Affiliations:** ^1^Department of Oral and Craniofacial Sciences, Faculty of Dentistry, University of Malaya, 50603 Kuala Lumpur, Malaysia; ^2^Department of Oral Biology and Biomedical Sciences, Faculty of Dentistry, MAHSA University Jenjarom, Malaysia; ^3^Department of Oral & Maxillofacial Clinical Sciences, Faculty of Dentistry, University of Malaya, 50603 Kuala Lumpur, Malaysia; ^4^Faculty of Bioresource and Food Industry, University Sultan Zainal Abidin, 22200, Terengganu, Malaysia; ^5^Institute of Agrobiotechnology, Universiti Sultan Zainal Abidin, 22200 Besut, Terengganu Darul Iman, Malaysia; ^6^Natural Medicine Research Centre, Universiti Islam Malaysia, 63000, Cyberjaya, Selangor Darul Ehsan, Malaysia

## Abstract

Goniothalamin (GTN) is an isolated compound from several plants of the genus* Goniothalamus*, and its anticancer effect against several cancers was reported. However, there is no scientific data about effects of its higher doses on internal organs. Accordingly, this study aimed to evaluate the acute and subacute effects of higher doses of GTN on the hematology, biochemistry, and histology of selected internal organs of male* Sprague-Dawley *rats. In acute study, 35 rats were distributed in 5 groups (n=7) which were intraperitoneally (IP) injected with a single dose of either 100, 200, 300, 400, or 500 mg/kg of GTN, while extra 7 rats serve as a normal control. In subacute study, 7 rats were IP-injected with a daily dose of 42 mg/kg of GTN for 14 days, while another 7 rats serve as a normal control group. The normal controls in both studies were IP-injected simultaneously with 2 ml/kg of 10% DMSO in PBS. At the end of both tests, rats were sacrificed to collect blood for hematology and biochemistry and harvest livers, kidneys, lungs, hearts, spleens, and brains for histology. During acute and subacute exposure, no abnormal changes were observed in the hematology, biochemistry, and histology of the internal organs. However, the 300, 400, and 500 mg/kg of GTN during acute exposure were associated with morbidities and mortalities. Ultimately, GTN could be safe up to the dose of 200 mg/kg, and the dose of 42 mg/kg of GTN was tolerated well.

## 1. Introduction

The knowledge of using the natural products to cure both human and animal diseases has been transmitted from a generation to another which was perfected with experience and gained wide acceptance in recent times [1, 2] in addition to the impression that natural plant medicines have fewer side effects [3, 4]. The World Health Organization (WHO) estimated that about 70-80% of the population has used traditional medicine in some way or another in many developed countries [5]. Similarly, there is also an increasing interest in herbal medicines among people in the developed countries [6]. However, safety of herbal medicines is still not sufficiently explored [5].

In fact, the use of herbal medicines attracts criticism largely due to the lack of scientific assessment of their toxicity since some of the herbal medicines have been found to be toxic [7, 8]. For such reason, the toxicity of herbal medicines should be evaluated particularly that some medicinal properties of such herbal medicines have already been confirmed [9]. In addition, toxicity studies are pivotal in determining the toxicity of a given substance using a selected animal model in the prediction of adverse effects, which are extrapolated to humans to determine the safety level of the substance used [10].

One of the widely used and studied plant derivatives is Goniothalamin (GTN) (a bioactive styrylpyrone derivative) that is isolated from* Goniothalamus species* within* Annonaceae* family, which is widely distributed throughout Asia and used as a medicinal plant [11–14]. As a matter of fact, several studies reported the cytotoxicity of GTN against many cancer cell lines [15–17]. In comparison with other anticancer agents (e.g., doxorubicin), GTN showed cytotoxicity against cancer cell lines but no toxicity against normal Chang liver cells [18]. Although several studies have reported the potent antiproliferative activity of GTN against several tumour cell lines* in vitro*, a little is known about its* in vivo* antitumor activity. A very recent study evaluated the effect of Celecoxib and GTN treatments on the morphological, hormonal, and inflammatory responses in the prostate anterior lobe in transgenic adenocarcinoma of the mouse prostate (TRAMP). The mice received 10 mg/kg oral dose of Celecoxib and 150 mg/kg oral dose of GTN. Cancer delay in the prostate anterior lobe was observed following both Celecoxib and GTN treatment with a GTN better treatment spectrum in the signalling pathways in the prostate microenvironment, mainly in Estrogen Receptor alpha [19]. Hence, there is a lack in information of the effective dosage of GTN against cancer to the dosage that causes toxicity and margin of safety (MOS). However, Vendramini et al, [20] evaluated the antitumor activity of GTN in solid Ehrlich tumor. Balb/C mice reported that intraperitoneal doses of 50, 100, and 300 mg/kg of GTN indicated no evidence of acute toxicity or weight loss in mice after 4 hours and the following 15 days [20]. However, the study did not provide the effect of GTN on hematological, biochemical, and histological parameters. In addition, there is insufficient data on acute and subacute exposure to higher doses of GTN in animals. Hence, this study aimed to investigate the histological, biochemical, and hematological changes associated with GTN administration on selected internal organs of male* Sprague-Dawley* rats.

## 2. Materials and Methods

### 2.1. Goniothalamin

Goniothalamin (GTN) was obtained from Dr. Abdul Manaf Ali, Faculty of Bioresource and Food Industry, University Sultan Zainal Abidin, Terengganu, Malaysia. The GTN compound was dissolved in phosphate-buffered saline and 10% DMSO and stored under 4°C.

### 2.2. Physicochemical Properties of GTN

**Scheme 1:**
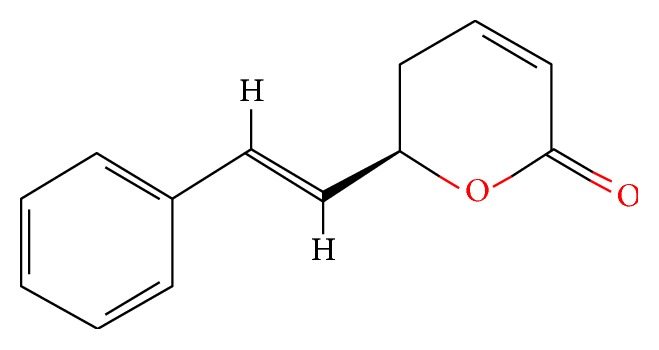
Molecular structure of Goniothalamin (GTN) [21].

It is a water insoluble solid crystalline powder with a molecular formula of GTN being C_13_H_12_O_2_, molecular weight 200.237 g/mol [21] (see Scheme 1).

### 2.3. Animals

Male* Sprague-Dawley *rats of 6 to 8 weeks old and an average weight of 200 - 230 g were purchased from Animal Experimental Unit (AEU) Faculty of Medicine (FOM), University of Malaya (UM), Malaysia. The rats were housed in plastic cages, three rats per cage, and maintained in the AEU for one week under standard conditions of temperature (25°C ± 2°C) and relative humidity (70% ± 5%) and 12-hour light-dark cycle. The rats were fed on standard rat pellets and allowed free access for tap water* ad libitum* throughout the experiments. The animal handling, from the beginning to the end of the study, was ethically conducted according to the agreed guidelines for the Animal Ethics Committee for Animal Experimentation, FOM, UM, Malaysia (Ethics No. 2016-190607/DENT/R/A). The maintenance and care of animals followed the guidelines of National Research Council [22].

### 2.4. Acute Toxicity Study

The experimental protocol was designed according to a previous study of Ahmed et al. [23]. A total of 35 rats were divided randomly into 5 groups (n= 7 rats) and each group received its specified treatment of GTN as a single intraperitoneal (IP) dose of either 100, 200, 300, 400, or 500 mg/kg, respectively. An extra group of rats (n=7) serve as a normal control group, which were simultaneously IP-injected with 2 ml/kg of the vehicle (10% of DMSO in PBS). After administration of the doses, all rats in the 6 groups were observed for 4 and 24 hours. At the end of the 24 hours of observation period, all rats were sacrificed. During the experiment, all observations for each rat in the 6 groups were systematically recorded and maintained as individual records. At the end of the experiment, the dead rats were counted in each group, and the lethal dose (LD: dose that kills 50% of animals) was calculated which was 420 mg/kg according to Miller and Tainter [24].

### 2.5. Subacute Toxicity Study

A total of 14 rats were used in this study and divided into two groups (n= 7). One group served as the experimental group while the other group served as the normal control. The rats in the experimental group received a single intraperitoneal daily dose of 42 mg/kg (i.e., 1/10 of the LD) of GTN compound for 14 days, while the rats in the control group were simultaneously IP-injected with 2 ml/kg of the vehicle (10% DMSO in PBS) [23]. The whole rats were observed continuously for 4 hours after dosing and then daily for a period of 14 days. The body weights of animals were recorded before the treatment and at the end of the experiment while food consumption and water intake were recorded daily.

### 2.6. Observation of the General Toxicity Signs

For both acute and subacute toxicity experiments, the rats were observed for the general toxicity signs such as effects on locomotion, behaviour (agitation, decreased activity, and somnolence), breathing, salivation, lacrimation, cyanosis, and death [25, 26]. At the end of both acute and subacute toxicity experiments, all the rats were anesthetized using an intraperitoneal injection of 80 mg/kg of ketamine and 7 mg/kg of xylazine (Troy laboratories PTY. Limited, Smithfield, Australia), and blood samples were collected via cardiac puncture for haematological and biochemical evaluations. In addition, an autopsy was carried out in all rats in the acute and subacute toxicity experiments, and kidneys, livers, lungs, hearts, spleens, and brains were harvested and preserved in 10% buffered formalin. Then organs were embedded in paraffin wax to be sectioned (4 *μ*m) and stained for the histopathological evaluation using Eosin and Hematoxylin stains. The histopathological evaluation in this study was performed using a light microscope (Nikon E50i). An experienced pathologist who was unaware of the experimental groups to which each section belonged conducted the analysis.

### 2.7. Statistical Analysis

Results were expressed as a mean ± standard deviation. The differences between groups of acute and subacute toxicity tests were determined by analysis of variance (one-way ANOVA) and independent* t*-test to compare a couple of variables, respectively. Differences in means were considered significant at* P *< 0.05.

## 3. Results

### 3.1. Acute Toxicity

#### 3.1.1. Clinical Observation of Acute Toxicity

No signs of toxicity or mortality were observed or recorded among rats, which were treated with 100 and 200 mg/kg GTN. However, eye secretion (within one hour of treatment), a reduction in mobility (without a response to stimuli), and tremors (after 2 hours of treatment) were the signs that were observed among 300 mg/kg of GTN-treated rats; however, all rats recovered after 4 hours of the treatment, and no mortality was recorded. In the 400 mg/kg GTN-treated group, only two rats suffered from tremors and breathing difficulty and then died within 4 hours of the experiment, while the remaining five rats showed eye secretion (after one hour of treatment), a reduction in mobility (without a response to stimuli), and tremors (within two hours of treatment), which recovered four hours later. All rats in the 500 mg/kg GTN-treated group died within four hours of the treatment after developing several signs of toxicity during the first hour of the treatment including tremors, breathing difficulty, Straub tail, opisthotonos, and scattered seizures during the first hour of treatment. Therefore, the rate of mortality was 0% among the rats which were treated with 100, 200, and 300 mg/kg GTN, while the rate of mortality among the 400 and 500 mg/kg GTN-treated rats was 29% and 100%, respectively.

### 3.2. Hematological Evaluation

Table 1 showed that all haematological parameters of 100, 200, 300, 400, and 500 mg/kg GTN-treated rats were not significantly different from those of rats in the control group.

### 3.3. Biochemical Evaluation

Table 2 showed no significant differences in serum creatinine, urea, albumin, globulin, total bilirubin, alkaline phosphatase, alanine aminotransferase, and aspartate aminotransferase of the 100, 200, 300, 400, and 500 mg/kg GTN-treated rats as compared with those of rats in the normal control group.

### 3.4. Histopathological Evaluation

**Figure 1:**
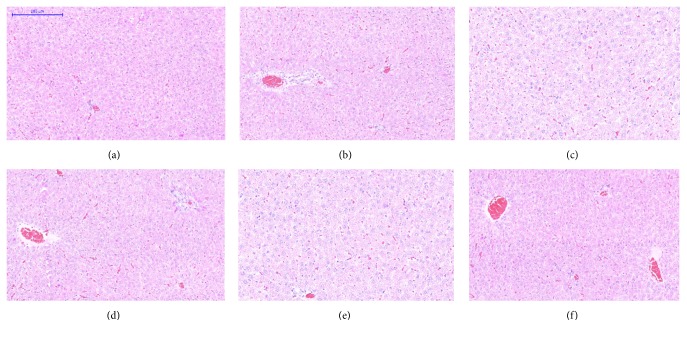
Liver histological sections of rats in acute toxicity study. (a) Normal control group; (b) 100 mg/kg GTN intraperitoneal-treated group; (c) 200 mg/kg GTN intraperitoneal-treated group; (d) 300 mg/kg GTN intraperitoneal-treated group; (e) 400 mg/kg GTN intraperitoneal-treated group; (f) 500 mg/kg GTN intraperitoneal-treated group (100×, H&E stained).

**Figure 2:**
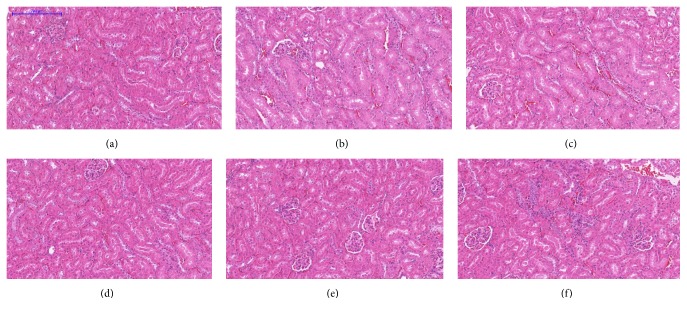
Kidney histological sections of rats in acute toxicity study. (a) Normal control group; (b) 100 mg/kg GTN intraperitoneal-treated group; (c) 200 mg/kg GTN intraperitoneal-treated group; (d) 300 mg/kg GTN intraperitoneal-treated group; (e) 400 mg/kg GTN intraperitoneal-treated group; (f) 500 mg/kg GTN intraperitoneal-treated group (100×, H&E stained).

**Figure 3:**
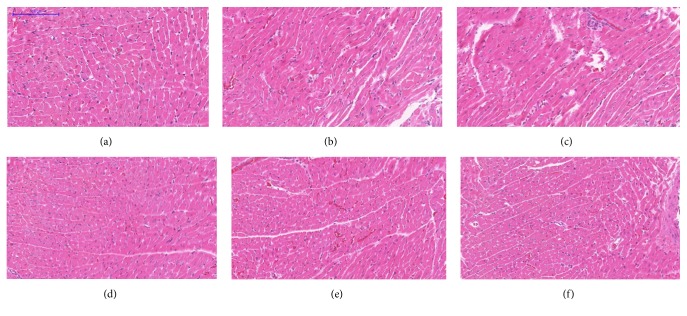
Heart histological sections of rats in acute toxicity study. (a) Normal control group; (b) 100 mg/kg GTN intraperitoneal-treated group; (c) 200 mg/kg GTN intraperitoneal-treated group; (d) 300 mg/kg GTN intraperitoneal-treated group; (e) 400 mg/kg GTN intraperitoneal-treated group; (f) 500 mg/kg GTN intraperitoneal-treated group (100×, H&E stained).

**Figure 4:**
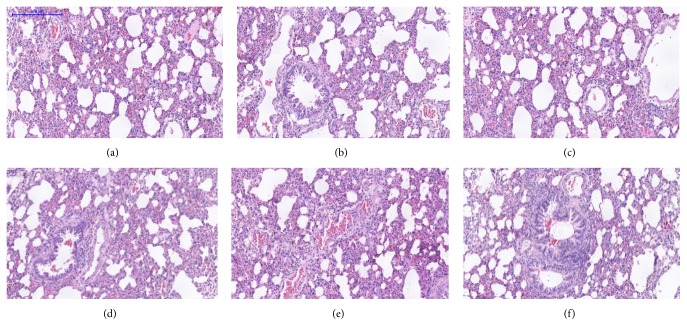
Lung histological sections of rats in acute toxicity study. (a) Normal control group; (b) 100 mg/kg GTN intraperitoneal-treated group; (c) 200 mg/kg GTN intraperitoneal-treated group; (d) 300 mg/kg GTN intraperitoneal-treated group; (e) 400 mg/kg GTN intraperitoneal-treated group; (f) 500 mg/kg GTN intraperitoneal-treated group (100×, H&E stained).

**Figure 5:**
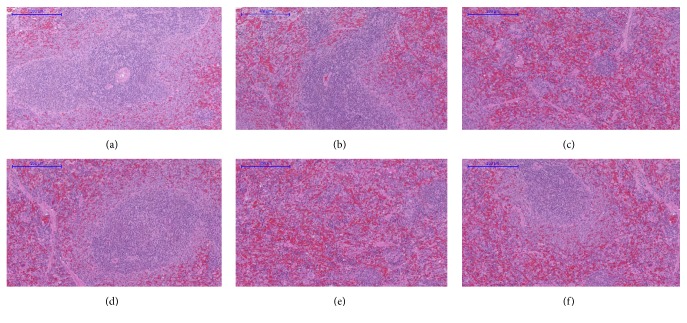
Spleen histological sections of rats in acute toxicity study. (a) Normal control group; (b) 100 mg/kg GTN intraperitoneal-treated group; (c) 200 mg/kg GTN intraperitoneal-treated group; (d) 300 mg/kg GTN intraperitoneal-treated group; (e) 400 mg/kg GTN intraperitoneal-treated group; (f) 500 mg/kg GTN intraperitoneal-treated group (100×, H&E stained).

**Figure 6:**
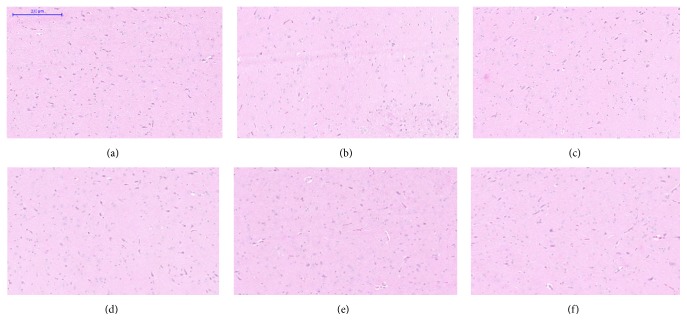
Brain histological sections of rats in acute toxicity study. (a) Normal control group; (b) 100 mg/kg GTN intraperitoneal-treated group; (c) 200 mg/kg GTN intraperitoneal-treated group; (d) 300 mg/kg GTN intraperitoneal-treated group; (e) 400 mg/kg GTN intraperitoneal-treated group; (f) 500 mg/kg GTN intraperitoneal-treated group (100×, H&E stained).

Microscopic examination of the tissue's sections of liver (Figure 1), kidney (Figure 2), heart (Figure 3), lung (Figure 4), spleen (Figure 5), and brain (Figure 6) of rats that were treated with 100, 200, 300, 400, and 500 mg/kg of GTN showed normal architectures with unnoticeable differences in the histological and cellular structures of all organs. In liver, the cellular structures of hepatocytes, sinusoids, and central vein were similar to those in control group. In the heart, the cellular structures of cardiac muscle cell and connective tissue were normal. In the lung, the cellular structures of bronchiole, alveoli, alveolar duct, and blood vessel were normal. Similarly, no abnormalities were observed in the spleen and brain of the rats following the GTN administration as compared with those of rats in the normal control group.

### 3.5. Subacute Toxicity

#### 3.5.1. Clinical Observation

No deaths were observed after 14 days of treatment of rats with 42 mg/kg GTN-treated group.

#### 3.5.2. Body Weight Changes

No statically significant changes were obtained between the body weight of the GTN-treated group and the normal control group (Table 3).

### 3.6. Absolute Weight of the Selected Organs

The absolute weights of the selected organs of the GTN-treated group were within the normal range and no significant differences were observed between the absolute weights of the selected organs of the GTN-treated group and those of the normal control group (Table 4).

### 3.7. Food Consumption and Water Intake of Rats

There were no significant changes in food consumption and water intake between the GTN-treated group and the normal control group (Table 5).

### 3.8. Haematological and Biochemical Evaluation

There were no significant differences in haemoglobin, white blood cells count, neutrophils percent, lymphocytes percent, monocytes percent, eosinophils percent, and basophils percent of the GTN-treated group as compared with those of rats in the normal control group (Table 6).

### 3.9. Biochemical Evaluation

A nonsignificant alteration could be detected in the serum creatinine, urea, albumin, globulin, alkaline phosphatase, alanine aminotransferase, and aspartate aminotransferase of the GTN-treated rats as compared with those of the rats in the normal control group (Table 7).

### 3.10. Histopathological Evaluation

**Figure 7:**
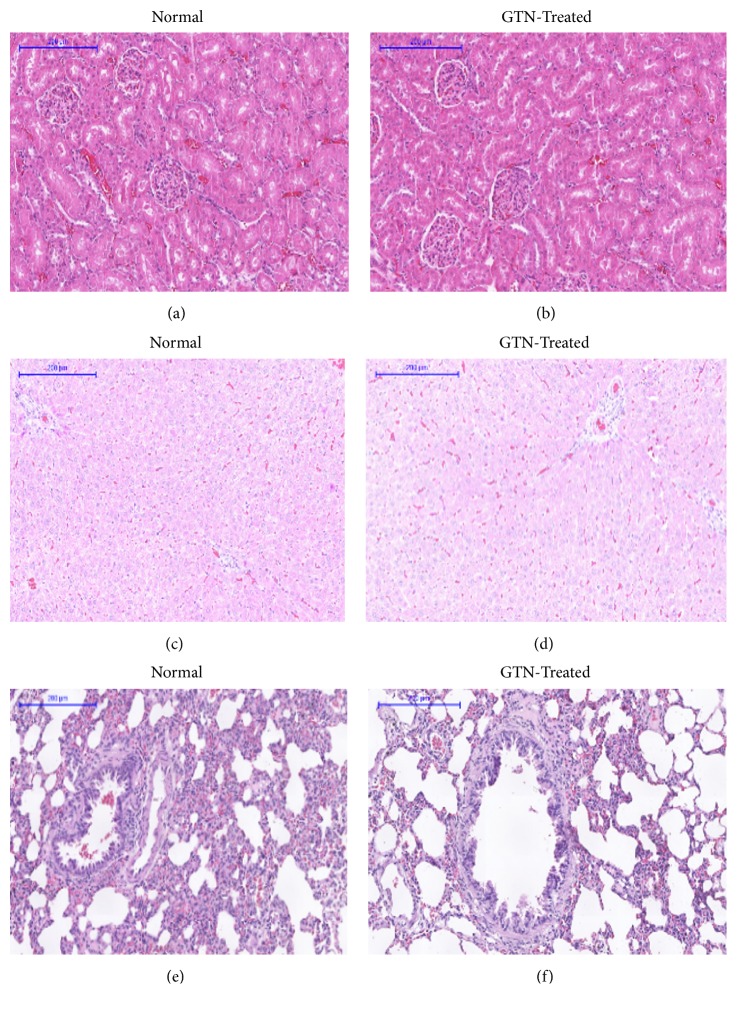
Histological sections of kidney, liver, and lung of rats in subacute toxicity. (a), (c), and (e) represent histological sections of kidney, liver, and lung of the rats in the normal control group, respectively. (b), (d), and (f) represent histological sections of kidney, liver, and lung of the rats intraperitoneally treated with 42 mg/kg of GTN, respectively (100×, H&E stained).

**Figure 8:**
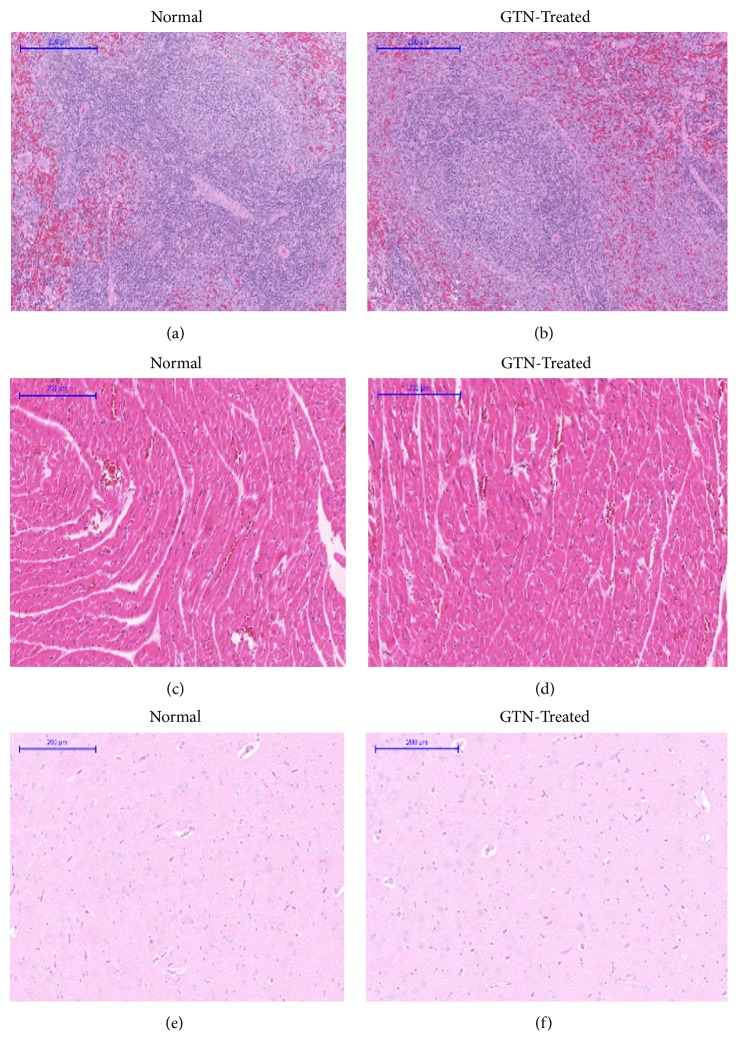
Histological sections of spleen, heart, and brain of rats in subacute toxicity. (a), (c), and (e) represent histological sections of spleen, heart, and brain of the rats in the normal control group, respectively. (b), (d), and (f) represent histological sections of spleen, heart, and brain of the rats intraperitoneally treated with 42 mg/kg of GTN, respectively (100×, H&E stained).

A histopathological evaluation was carried out to confirm the biochemical findings as shown in Table 7 and to identify any structural changes. Light microscopic examination of the vital organs including liver, kidney, heart, lung, spleen, and brain of the rats in all the GTN-treated and control groups for subacute toxicity (Figures 7 and 8) did not reveal any gross pathological lesions. The photomicrographs of the liver and kidney of the control and GTN-treated groups showed normal morphological architecture. Under microscopic examination, the liver of GTN-treated animals showed normal cellular architecture and binucleation and was without any distortions similar to the control group. Furthermore, signs of injury, necrosis, congestion, fatty acid accumulation, or hemorrhagic regions around the central vein or sinusoids of the liver were not observed. The hepatocytes arranged in cords were clearly visible. The cross-section of the liver showed no lyses in the blood cells, or infiltration of neutrophil, lymphocyte, or macrophage in the subacute oral toxicity. As for the kidneys, histologically there was no morphological change for the GTN-treated group. The appearance of the glomerular architecture was normal similar to the control groups. The glomeruli, distal, and proximal tubules in the kidney appeared normal in both groups. In addition, there was no interstitial and intraglomerular congestion or tubular atrophies. All the nephron cells were normal and showed clearly visible nucleoli with no degeneration, bleeding, necrosis, or infiltration with lymphocytes. In both the control and GTN-treated rats, the heart showed normal cardiac muscle fibers and lungs showed normal alveolar structure with no treatment-related inflammatory response. Similarly, normal structure and histology of the spleen was also observed in all the rats. Thus, the histopathological evaluations of the selected organs did not reveal any morphological abnormalities that could be attributed to the administration of GTN compound to the rats.

## 4. Discussion

Goniothalamin (GTN) active compound is a natural compound derived from a plant called* Goniothalamus macrophyllus* distributed in the rainforest of Peninsula, Malaysia, Sabah, and Sarawak and also in other Southeast Asian countries. Reviewing the literature indicated a lack of previous toxicity studies on GTN except one study that was reported by Mosaddik and Haque [27] who isolated GTN from* Bryonopsis laciniosa Linn*. In their study, GTN was administered intraperitoneally to rats in a dose of 300 *μ*g/day for 14 days indicating no toxic effects [27]. Nonetheless, the dose that was administered to rats in their work was very low which could make the toxicity of higher doses of GTN and its lethal dose unpredictable. Accordingly, the current study was conducted to evaluate the effects of higher doses of GTN on the hematological and biochemical parameters as well as the direct histological effects of such doses on selected internal organs of rat models.

The significant variation in the rat behavior suggests an alteration in the general state of the animal [28]. In the acute toxicity study, the findings indicated that the toxicity of GTN increased with the increase in the dose particularly the 400 and 500 mg/kg. Accordingly, the acute study enabled us to determine the lethal dose of GTN which was determined to be 420 mg/kg. Conversely, the hematological markers showed no significant difference in all the treated rats with different doses of GTN. However, death incidence has been still obscure even after autopsy, particularly the histological examination of organs showed normal architectures in the livers, kidneys, hearts, lungs, and spleens of the treated rats with the different doses of GTN, which needs further investigation in the future. Perhaps the rat death in the 400 and 500 mg/kg groups could mean that GTN has a central action that interferes with breathing capacity which leads to breathing difficulties and then causes death.

In the subacute toxicity study, our findings demonstrated a nonsignificant difference in the body weights of rats which were treated with the different doses of GTN as compared with those of rats in the normal control group indicating no organic changes [28]. In addition, no significant difference could be detected in food consumption and water intake of rats that were treated with the different doses of GTN as compared with those of rats in the normal control group. Maybe the nonsignificant difference in food and water intake could explain the nonsignificant differences in body weight. Other anticancer agents such as Cisplatin may reduce the body weight of rats. Atasayar et al. [29] reported that a single dose of 7.5 mg/kg of Cisplatin injected to rats significantly decreased the body weight [29]. Similarly, hematological parameters of the rats which were treated with the different doses of GTN showed no significant differences as compared with those of rats in the normal control group, which could indicate no abnormalities in the metabolic processes, injury or lesion, deprivation, and drug-related stress [30]. Therefore, it could be assumed that the tested doses of GTN did not cause any alterations of a biological importance in the hematological parameters. In contrast to the present study results, Wood and Hrushesky [31] had treated rats with 2 mg/kg of Cisplatin for 4 weeks and observed that the hemoglobin value was only 5.9 g/dl and the white blood cells and platelets values were also reduced to the lower normal range [31].

The liver and kidney are important organs, which are responsible for the metabolism, detoxification, storage, and excretion of xenobiotics and their metabolites and are susceptible to damage by external substances [32]. However, the liver is a complex organ which is comprised from several cell types performing various functions, and those cells can be damaged by different pathways [33]. Once the hepatic cell membrane is damaged, the cytosol enzymes are released into the blood, such as aspartate aminotransferase (AST), alanine aminotransferase (ALT), and alkaline phosphatase (ALP) [34]; however, both AST and ALT are intracellular enzymes of which appearance in the blood is an indicative of a cellular damage [35]. Therefore, their determination in serum could be used to assess any incident organic damage, particularly that there are established normal ranges of universal markers for the detection of organic damage [30]. Otherwise, there is no single biochemical marker that can be relied on as a universal test of liver damage [33], although AST and ALT are the serum enzymes that have been shown to be the most effective and sensitive indicators of hepatocellular injury [34]. Unfortunately, AST also can exist in many organs including the heart and muscles; therefore, its release is not specific for acute liver diseases [36]. Unlike AST, ALT is primarily found in the liver [35]. Regarding the serum level of ALP, this enzyme is ubiquitous in several organs including liver, bone, kidney, intestine, and placenta and its exact role differs from one tissue to another; however, the major elevation of serum ALP is associated with liver and bone dysfunctions [37] in addition to the fact that ALP is rapidly elevated due to the impairment in bile flow or expansive lesions of different types [38]. Moreover, the absence of bone disease could reflect that the elevated ALP levels could be an indicator of biliary tract dysfunction or in response to cholestasis [39]. Our findings of acute and subacute studies indicated nonsignificant differences in serum levels of AST, ALT, and ALP in rats which were treated with 100, 200, 300, 400, or 500 mg/kg of GTN as compared with those of rats in the normal control group. The liver is the main metabolizing and detoxing organ for drugs making liver a common target of xenobiotic damage in addition to the fact that several apparently safe drugs may occasionally produce severe liver-related adverse reactions [40] which stand behind the most common cause of withdrawal of drugs from the market [41]. However, the adverse drug reactions in the liver can be difficult to be diagnosed because drugs that induce liver damage can mimic almost any type of hepatobiliary disease [40]. On the other hand, our findings of liver histological sections of all rats in the acute and subacute studies indicated normal architectures without any lesion, although it is almost impossible to differentiate histologically between liver damage caused by drugs and that produced spontaneously by a disease, since drug-induced damage includes virtually all types of known acute and chronic liver damage [40]. Our findings of the normal levels of ALT and AST were inconsistent with the reported findings by Palipoch and Punsawad [42] who found that injecting rats with a single dose of Cisplatin of 10, 25, and 50 mg/kg for 24, 48, 72, 96, and 120 hours could increase the serum levels of ALT and AST due to induction of hepatorenal oxidative stress [42]. In addition, our findings of normal levels of ALP, AST, and ALT because of injecting rats with 100, 200, 300, 400, and 500 mg/kg of GTN were in contrast to the reported serum increased levels of such enzymes due to the intraperitoneal injection of 12 mg/kg of Cisplatin to rats [43].

To evaluate the renal function of the rats in the present study, urea and creatinine levels were measured. As it is well known the kidney performs three main functions including elimination of toxic substances that are produced during metabolism, regulation of internal liquid medium hemostasis, and production of hormones which could be used to assess the renal status [35]. When the kidneys fail, acutely or chronically, the end product of nitrogen metabolism builds up, increasing nonprotein nitrogen levels which can be expressed in the form of elevation of blood urea nitrogen and serum creatinine [35]. Accordingly, renal function in blood can be evaluated through the determination of urea as the end product of protein metabolism which is formed in the liver from ammonia and later eliminated by the kidney [44] or through the determination of serum creatinine, which is a product of spontaneous nonenzymatic cleavage of phosphocreatine in the muscle, being excreted unchanged through the kidney. In most species this occurs only by filtration, plasma creatinine levels being taken as a measure of glomerular filtration rate. However, some extrarenal factors such as massive myonecrosis or prolonged strenuous exercise may temporarily affect the level of creatinine [11]. Our findings indicated that serum urea and creatinine levels showed no statistically significant difference in both acute and subacute studies of all doses of GTN as compared with those of rats in the normal control group; however, plasma creatinine concentration is not that sensitive indicator of renal dysfunction unless the flow rate of glomerular filtrate has fallen below 50% [35].

The absolute weights of rats' internal organs (livers, kidneys, hearts, lungs, and spleens) after the subacute studies were also in the normal range and without apparent significant differences. Moreover, the histological examination of the histological sections of livers, kidneys, hearts, lungs, and spleens indicated normal architectures with no lesions which could be in the support of the normal hematological and biochemical parameters indicating that GTN was nontoxic. However, the absolute safety of GTN is in terms of one-day (acute study) or 14-day (subacute study) investigation of the hematological, biochemical, and histological parameters in terms of the duration of the acute toxicity or subacute toxicity which was not adequate to induce an abnormal significant increase in the hematological and biochemical parameters or even abnormalities in the histological sections of the different organs. Therefore, it is recommended to prolong the duration of study of GTN toxicity particularly that GTN was suggested to exert an anticancer activity.

The histological findings of the current study of acute and subacute toxicity demonstrate that GTN shows no renal tubular or tubular necrosis. Comparing this finding to other anticancer agents, GTN showed high renal tolerance. For instance, many previous studies reported that Cisplatin is nephrotoxic at the lower end doses of the therapeutic range for clinical use. Acute tubular necrosis, cystic tubular dilatation, tubular regeneration, and renal tissue inflammation were reported when Cisplatin was administered into rats at a dose 7.5 mg/kg of body weight [45]. Other researchers administered a low dose of Cisplatin (0.4 mg/kg) into rats daily for 8 weeks and it resulted in an irreversible kidney injury of acute tubular necrosis, a severe atrophy of glomerulus, and marked dilation of proximal convoluted tubules [46]. Recent chemotherapeutic agents are often associated with nephrotoxicity complications leading to minimizing the therapeutic doses and thus to limit the antitumor effect of those agents [47]. Moreover, in a recent study, Ribeiro et al. [48] investigated the effect of obesity on the nephrotoxic effects of 20 mg/ kg of body weight of Cisplatin in mice. They found that Cisplatin causes acute renal injury especially to obese mice [48]. The possible explanation for undetectable renal toxicity of GTN could be due to its anti-inflammatory activity. Many previous studies reported the anti-inflammatory activity of GTN. After being isolated in 1972, many subsequent studies have reported different GTN properties such as anti-inflammatory, gastroprotective, cytotoxicity against cancer cell lines, and apoptosis induction [15, 49–52]. Regarding the liver, although hepatotoxicity is not considered as much a dose-limiting factor as nephrotoxicity, previous studies reported that high doses of Cisplatin also induce hepatotoxicity [53].

The present study findings showed that GTN has a higher safety range with a remarkably high safe upper limit in comparison with already clinically established anticancer agents such as Cisplatin, tamoxifen, doxorubicin, taxol, and 5-Fluorouracil (5-FU) [54, 55]. Similar to tamoxifen or taxol drugs, GTN showed cytotoxicity against ovarian cancer cell lines (Caov-3) but is toxic against normal kidney cells (MDBK) such as what was found in the case of tamoxifen or taxol agents [18]. Another study compared GTN toxicity with the chemotherapeutic drug doxorubicin against normal Chang liver cells; a much lower toxicity of GTN on those cells was reported [11]. Taking the above into account, GTN may be considered as a promising chemotherapeutic agent.

## 5. Conclusions

Intraperitoneal administration of GTN into rats produced no changes in the hematological parameters and the biochemical markers of hepatic and renal function neither during acute nor subacute exposure. However, GTN was safe up to the dose level of 200 mg/kg after acute intraperitoneal exposure to GTN, while the dose of 42 mg/kg of GTN for 14 days was well-tolerated by rats.

## Figures and Tables

**Table 1:** Haematological evaluation for acute toxicity.

	Control	100mg/kg	200mg/kg	300mg/kg	400mg/kg	500mg/kg
Hemoglobin (g/dL)	16.52 ± 1.22	15.46 ± 1.12	15.62 ± 1.31	15.12 ± 2.21	16.13 ± 1.46	15.11 ± 2.08
White blood cells (10^9^/L)	6.33 ± 0.32	6.11 ± 0.52	6.61 ± 0.58	5.99 ± 1.14	6.47 ± 053	5.71 ± 1.21
Neutrophils (10^9^/L)	18.22 ± 0.23	18.41 ± 0.31	17.17 ± 2.35	17.22 ± 3.15	17.30 ± 0.50	18.33 ± 1.21
Lymphocyte (10^9^/L)	78.21 ± 2.26	80.16 ± 5.31	76.86 ± 2.17	80.66 ± 3.11	79.23 ± 1.11	76.57 ± 1.55
Monocytes (10^9^/L)	0.89 ± 0.11	1.00 ± 0.10	0.87 ± 0.12	0.87 ± 0.21	0.85 ± 0.12	0.88 ± 0.10
Eosinophils (10^9^/L)	3.23 ± 3.68	3.13 ± 2.79	3.41 ± 3.21	3.22 ± 2.31	3.11 ± 2.45	3.51 ± 2.55
Basophils (10^9^/L)	0.01 ± 0.21	0.01 ± 0.11	0.01 ± 0.02	0.01 ± 0.07	0.01 ± 0.10	0.01 ± 0.06

The results are expressed as the mean ±Standard error, (n=7). No significant difference could be detected between the different groups vs. the control group; Control, normal control group; 100 mg/kg, 100 mg/kg GTN- intraperitoneal treated group; 200 mg/kg, 200 mg/kg GTN- intraperitoneal treated group; 300 mg/kg, 300 mg/kg GTN- intraperitoneal treated group; 400 mg/kg, 400 mg/kg GTN- intraperitoneal treated group; 500 mg/kg, 500 mg/kg GTN- intraperitoneal treated group.

**Table 2:** Biochemical evaluation for acute toxicity.

	Control	100mg/kg	200mg/kg	300mg/kg	400mg/kg	500mg/kg
Creatinine (*µ*mol/L)	25.22 ± 1.13	25.13 ± 1.11	24.00 ± 2.15	24.52 ± 2.14	25.00 ± 1.22	26.24 ± 1.14
Urea (mmol/L)	5.95 ± 0.33	5.60 ± 0.15	5.87 ± 0.13	5.90 ± 0.21	5.91 ± 0.32	5.85 ± 0.15
Albumin (g/L)	35.9 ± 3.2	34.6 ± 3.7	34.2 ± 7.8	34.3 ± 3.2	33.5 ± 5.3	33.2 ± 4.3
Globulin (g/L)	23.2 ± 2.8	21.5 ± 1.8	22.3 ± 3.1	22.6 ± 2.9	21.2 ± 4.2	21.3 ± 3.9
Total bilirubin (*µ*mol/L)	2.0 ± 0.8	2.1 ± 0.7	2.0 ± 0.5	1.9 ± 0.4	1.9 ± 0.7	2.1 ± 0.9
Alkaline phosphatase (U/L)	432 ± 11	424 ± 29	431 ± 26	422 ± 13	420 ± 14	435 ± 17
Alanine aminotransferase (U/L)	118 ± 31	119 ± 11	116 ± 21	120 ± 10	117 ± 18	115 ± 12
Aspartate aminotransferase (U/L)	153 ± 56	155 ± 32	153 ± 21	152 ± 42	155 ± 23	152 ± 32

The results are expressed as the mean ±Standard error, (n=7). No significant difference could be detected between the different groups vs the control group; Control, the normal control group; 100 mg/kg, 100 mg/kg GTN- intraperitoneal treated group; 200 mg/kg, 200 mg/kg GTN- intraperitoneal treated group; 300 mg/kg, 300 mg/kg GTN- intraperitoneal treated group; 400 mg/kg, 400 mg/kg GTN- intraperitoneal treated group; 500 mg/kg, 500 mg/kg GTN- intraperitoneal treated group.

**Table 3:** Rats body weight for sub-acute toxicity.

	0 days	Week 1	Week 2
Control	250.25 ± 4.13	267.19 ± 5.11	288.22 ± 4.19
GTN-treated	245.8 ± 8.11	263.8 ± 6.13	284.8 ± 9.08

The results are expressed as the mean ±Standard error, (n=7). No significant difference could be detected between the GTN-treated group vs the control group. Control, the normal control group; GTN-treated, 42 mg/kg intraperitoneal GTN-treated group.

**Table 4:** Absolute weight of organs during subacute toxicity.

	Control	GTN-treated
Kidney	0.85 ± 0.02	0.87 ± 0.10
Liver	3.62 ± 0.11	3.55 ± 0.12
Brain	2.26 ± 0.12	2.25 ± 0.33
Lung	0.46 ± 0.02	0.45 ± 0.04
Heart	0.38 ± 0.02	0.37 ± 0.01
Spleen	0.27 ± 0.02	0.28 ± 0.01

The results are expressed as the mean ± Standard error, (n=7). No significant difference could be detected between the GTN-treated group vs the control group. Control, the normal control group; GTN-treated, 42 mg/kg intraperitoneal GTN-treated group

**Table 5:** Food consumption and water intake of rats during subacute toxicity.

	Control		GTN-treated	
	Food	Water	Food	Water
Week 1	73.34 ± 3.23	92.54 ± 4.13	75.32 ± 1.52	91.22 ± 1.02
Week 2	82.62 ± 1.34	93.14 ± 2.12	80.12 ± 1.42	92.31 ± 2.11

The results are expressed as the mean ± Standard error, (n=7). No significant difference could be detected between the GTN-treated group vs the control group. Control, the normal control group; GTN-treated, 42 mg/kg intraperitoneal GTN-treated group.

**Table 6:** Hematological evaluation for subacute toxicity.

	Control	GTN-treated
Hemoglobin (g/dL)	155.21 ± 0.76	154.11 ± 1.30
White blood cells (10^9^/L)	6.57 ± 0.21	6.73 ± 0.14
Neutrophils (10^9^/L)	9.13 ± 1.23	10.10 ± 2.11
Lymphocytes (10^9^/L)	77.22 ± 26.44	79.39 ± 21.22
Monocytes (10^9^/L)	2.21 ± 0.21	2.33 ± 0.14
Eosinophils (10^9^/L)	0.89 ± 0.90	0.91 ± 0.80
Basophils (10^9^/L)	0.01 ± 0.00	0.01 ± 0.15

The results are expressed as the mean ± Standard error, (n=7). No significant difference could be detected between the GTN-treated group vs the control group. Control, the normal control group; GTN-treated, 42 mg/kg intraperitoneal GTN-treated group.

**Table 7:** Haematological evaluation of GTN-treated sub-acute toxicity.

	Control	GTN-treated
Creatinine (*µ*mol/L)	25.22 ± 1.13	26.24 ± 1.14
Urea (mmol/L)	5.95 ± 0.33	5.85 ± 0.15
Albumin (g/L)	35.9 ± 3.2	33.2 ± 4.3
Globulin (g/L)	23.2 ± 2.8	21.3 ± 3.9
Total bilirubin (*µ*mol/L)	2.0 ± 0.8	2.1 ± 0.9
Alkaline phosphatase (U/L)	13.9 ± 1.1	14.2 ± 0.5
Alanine aminotransferase (U/L)	118 ± 31	115 ± 12
Aspartate aminotransferase (U/L)	99.5 ± 5.6	100 ± 7.1

The results are expressed as the mean ± Standard error, (n=7). No significant difference could be detected between the GTN-treated group vs the control group. Control, the normal control group; GTN-treated, 42 mg/kg intraperitoneal GTN-treated group.

## Data Availability

The data used to support the findings of this study are available from the corresponding author upon request.
